# Real-time monitoring of rhizosphere nitrate fluctuations under crops following defoliation

**DOI:** 10.1186/s13007-021-00713-w

**Published:** 2021-01-30

**Authors:** Nicola M. Capstaff, Claire Domoney, Anthony J. Miller

**Affiliations:** Department of Metabolic Biology, John Innes Centre, Norwich Research Park, Norwich, NR4 7UH UK

**Keywords:** Alfalfa, Forage crops, Grass, Leaching, *Lolium perenne*, Management practices, *Medicago sativa*, Nitrate, Rhizosphere, Soil profile

## Abstract

**Background:**

Management regime can hugely influence the efficiency of crop production but measuring real-time below-ground responses is difficult. The combination of fertiliser application and mowing or grazing may have a major impact on roots and on the soil nutrient profile and leaching.

**Results:**

A novel approach was developed using low-cost ion-selective sensors to track nitrate (NO_3_^−^) movement through soil column profiles sown with the forage crops, *Lolium perenne* and *Medicago sativa*. Applications of fertiliser, defoliation of crops and intercropping of the grass and the legume were tested. Sensor measurements were compared with conventional testing of lysimeter and leachate samples. There was little leaching of NO_3_^−^ through soil profiles with current management practices, as monitored by both methods. After defoliation, the measurements detected a striking increase in soil NO_3_^−^ in the middle of the column where the greatest density of roots was found. This phenomenon was not detected when no NO_3_^−^ was applied, and when there was no defoliation, or during intercropping with *Medicago*.

**Conclusion:**

Mowing or grazing may increase rhizodeposition of carbon that stimulates soil mineralization to release NO_3_^−^ that is acquired by roots without leaching from the profile. The soil columns and sensors provided a dynamic insight into rhizosphere responses to changes in above-ground management practices.

## Background

Grassland comprises nearly 12% of earth’s organic matter, much of which is belowground in roots and soil organic matter [1, 2]. Natural grasslands and forage crops are carbon sinks, important in the context of increasing atmospheric CO_2_ levels if they are properly managed [3, 4]. Furthermore, the global demand for food protein is increasing each decade and is estimated as 110 ± 7% each year [5], satisfying this need requires increased use of nitrogen (N) fertilisers [6]. The Haber–Bosch process fixes atmospheric N to make fertiliser and uses 1–2% of the world’s energy, and 3–5% of its natural gas expenditure [7]. Decreasing the high rates of N fertiliser use for animal farming and forage crop production is an important target, especially in high-intensity temporary grasslands. Excess N leaches into water supplies causing eutrophication of aquatic environments [8]. For example, in the UK forage grass and legume crops are predicted to have high leaching rates to the environment with this problem exacerbated when crops are cultivated in sandy soils [9]. It is estimated that 60% of applied N fertiliser may be lost through leaching, run-off, denitrification and consumption by microbial populations [10, 11]. N leaching can contaminate human drinking water especially in ground water supplies and may result in decreased life expectancy [12, 13]. In addition, N emissions from grassland and animal production contribute to climate change [14, 15].

Plants take up N from soil in a variety of forms, principally as NO_3_^−^ and ammonium, but also as amino acids and other organic N compounds [16, 17]. Many forage grasses, including *Lolium perenne*, preferentially uptake NO_3_^−^ [18], although the level of NO_3_^−^ can greatly vary within a field, even for plots metres apart [19, 20]. The ions mobility gives it a higher leaching potential than most other forms of soil N [21]. Repeated cutting and removal of the above ground crop is an essential part of forage management and the impact of defoliation has mostly been studied in hydroponically grown plants. In axenic culture defoliation of grass was shown to stimulate the release of carbon compounds from roots [22]. During the first seven days following defoliation of field grown ryegrass, decreases in biomass and N-content of stubble and roots were observed, concomitant with mobilization of N to shoots [23]. Below ground studies have shown that the C release after defoliation of grass can increase rhizosphere N mineralization rates after just 72 h [24] but decrease the rate organic matter breakdown [3]. In bi-cropping systems with legumes and grasses, de-topping the legume was shown to increase N concentration in the grass with more transfer of ^15^ N [25].

Soil N measurements are difficult to conduct and they can be unreliable and inconsistent, both across field samples and between testing methods [26]. Soil N estimates have long depended on soil core extracts, or porous ceramic cup or lysimeter samples being sent for later laboratory analysis [27]. Soil and soil water extract analysis is performed using conventional testing methods that include potassium chloride extraction alongside direct analysis of soil water using chromatography and colorimetric tests such as the Griess–Ilosvay reaction [28, 29]. Although these conventional testing methods have been used for decades, there are problems in their standardisation, use, and cost. Samples must be taken routinely and stored for testing, costing time and money. Subsequent N mineralization in soils is known to be affected by abiotic conditions [30], including pH [31], temperature [32], moisture [33], and soil texture and chemistry [31]. Therefore, laboratory analysis can be significantly different from actual real-time soil levels. This is exacerbated when there is high intra- and inter- variation in field soil N levels, as well as variation across seasons and spatially across depths [19, 34]. These conventional testing methods are labour-intensive and expensive and not conducive for addressing the aims of precision agriculture [35].

**Fig. 1:**
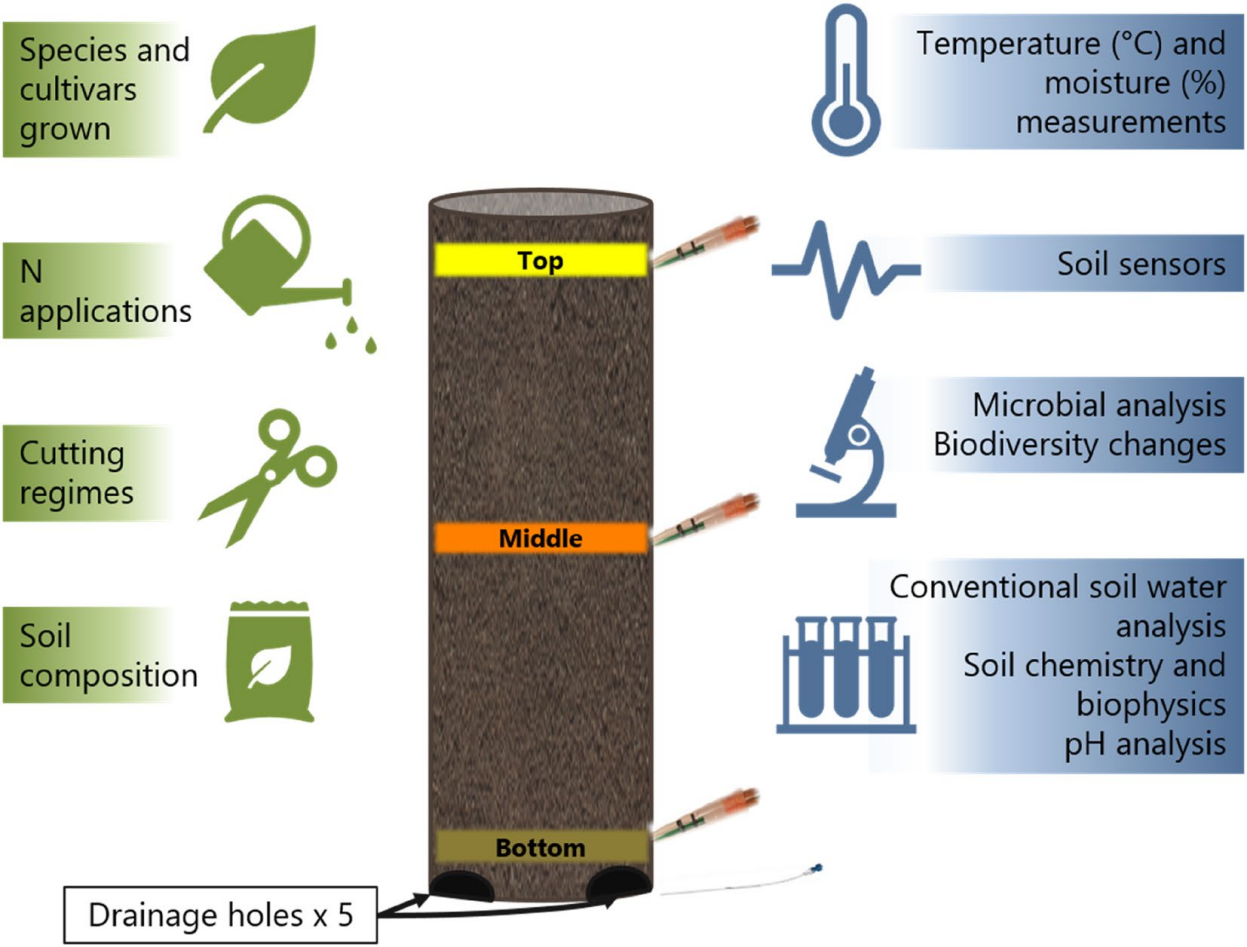
Generalised schematic diagram of column for NO_3_^−^-selective sensor experiments. Soil columns have dimensions of height = 50 cm and inside diameter = 15.4 cm. Possible experimental regime changes are marked in green, with the different assays possible shown in blue. Holes are available at three different levels for placement of the NO3−-selective sensors (shown as tip sensor photo symbol), at the top 1 cm (yellow), the bottom 1 cm (brown), and midway between (orange). Drainage holes were located at the bottom of the column, where soil water can be sampled using a lysimeter indicated with photo symbol of mini suction 10 Rhizon SMS lysimeters (Rhizosphere Research Products B.V., Wageningen, The Netherlands). Note microbial analysis was not performed in this work

Ion-selective electrodes can provide an alternative method for measuring *in-situ* real time soil NO_3_^−^ levels [36–38]. They may be used at different depths in the field to provide soil profile information or used in soil columns. Soil column experiments are used to gain accurate information on the interaction of roots and soil without the complications of field trials. They can be used to screen root rhizosphere systems and how these can interact [39]. Although soil columns are an artificial system, they are superior to media-based or hydroponic systems as their microbiology is more akin to that in nature [40]. Media-based and hydroponic systems lack soil structure and are suboptimal for studying root growth and nutrient uptake for N fertiliser experiments [41]. Soil experiments are a more representative model system for field interactions but studying soil profile behaviour under a crop is difficult. Use of soil columns can be a good compromise system. Figure 1 illustrates how soil columns can be used, particularly for studying aspects of forage crop cultivation. Conventional sampling methods and testing can also be used alongside NO_3_^−^-selective sensors in column experiments. These experiments can include testing at different depths, with experimental replicates carried out with reasonable practical ease within a convenient timescale, providing meaningful data relating to field systems.

Here we describe how soil N changes were measured using NO_3_^−^-selective sensors in response to cultivation of *L. perenne* in soil columns. Three depths of the soil NO_3_^−^ profile were assessed following different management practices. Management practices included varying N availability in the form of NO_3_^−^ application, defoliation of plant vegetative tissue to simulate crop harvest, and intercropping with the legume *Medicago sativa* (commonly called lucerne or alfalfa). As NO_3_^−^-selective sensors respond to changes in soil water nitrate concentration, we tested for their agreement to conventional soil water testing methods of leachate from column drainage holes. Columns were used to simulate the environment in the field and this approach provided data for forage crop agriculture on how management practices can influence soil N levels and the potential for fertiliser leaching.

## Results and discussion

### ***Soil column NO***_***3***_^***−***^*** profiles vary with management practices***

**Fig. 2:**
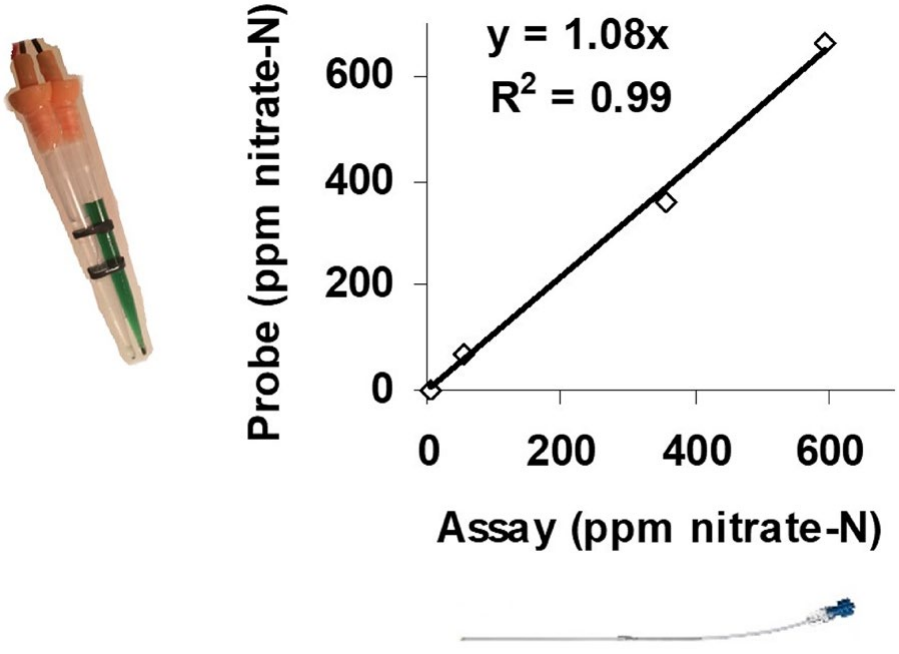
NO_3_^−^-selective sensors readings correlated with conventional soil water NO_3_^−^ extraction analysis assay results collected using a mini-suction lysimeter (see *Materials and Methods* for details). The same soil sample was treated with a range of nitrate concentrations each in four different pots and then measured for NO_3_^−^–N as ppm using both NO_3_^−^-selective sensors and suction lysimeter water samples assayed using a conventional Griess assay. NO_3_^−^–N ppm are the units most used by soil scientists; the sensors were calibrated to give outputs in mM too. The graph shows strong agreement between measurements (R^2^ = 0.99)

We began by comparing the soil nitrate measurements with the nitrate concentration measurements collected using mini-suction lysimeters (Fig. 2). There was an excellent correlation between data obtained using the two types methods in the same soil (Fig. 2). Soil N changes in columns were measured in response to the cultivation schemes listed in Table 1. These columns were termed ‘Monocrop 1 to 3′, with one column having no plants with a NO_3_^−^ application at day 0, ‘No crop’. ‘Monocrop 1′, had a dH_2_O application at day 0, ‘Monocrop 2′ had NO_3_^−^ application at day 0, and ‘Monocrop 3′ had NO_3_^−^ application plus vegetative tissue defoliation biomass measurement at day 28, representing the industry standard for regular cuts every 4–8 weeks.

**Table 1:**
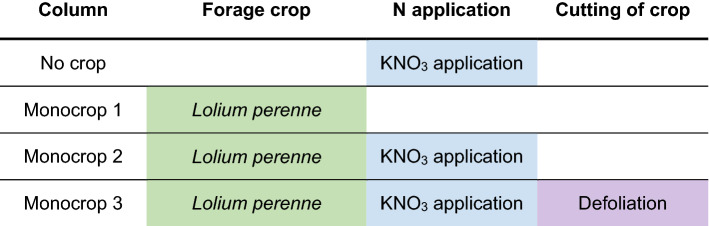
Column set-up for the *Lolium perenne* monocropping experiment

Columns were managed as colour coded and as follows; green for planting with 100% *L. perenne* seedlings, cv. Aber Magic, at seeding rate of 43.7 kg ha^−1^; blue for day 0 nitrate application with KNO_3_ treatments equivalent to 57 kg ha^−1^; purple for day 28 total aboveground vegetative defoliation with biomass measurement. All columns with crops growing were cut on day 56 for biomass totals, and in the case of ‘Monocrop 3′ this was added to the day 28 measurement

**Fig. 3:**
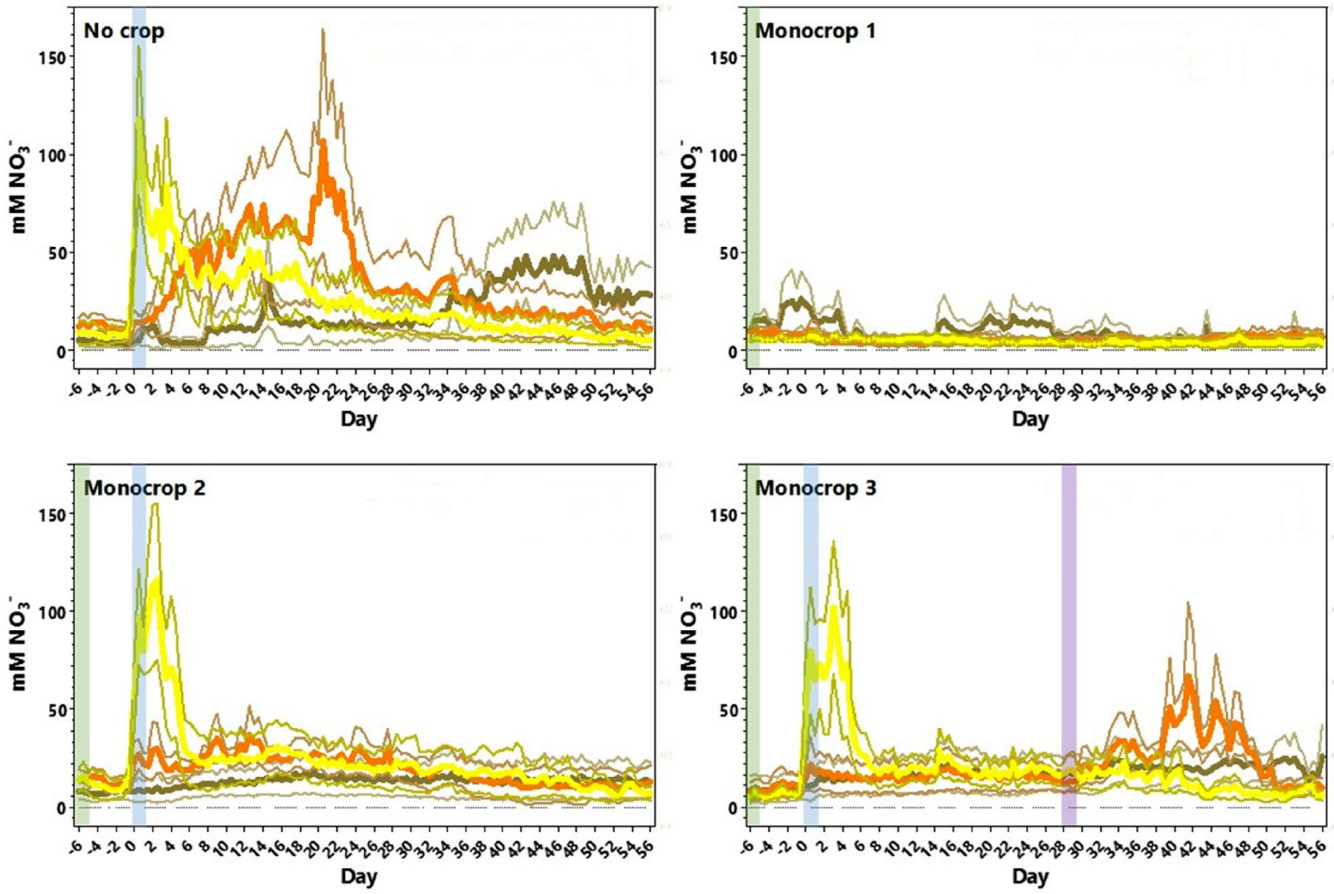
*Lolium perenne* monocrop column experiment NO_3_^−^-selective sensor data. Mean data (4 columns) are shown for each treatment as indicated in Table 1 as the thickest coloured lines. NO_3_^−^-selective sensor data are shown for top (yellow), middle (orange), and bottom (brown) levels of the columns. Data is the 12 h average of four experimental replicates in GraphPad Prism 7 (GraphPad Software Inc.), with standard error of the mean indicated with thinner lines of the same colour. Overlayed coloured vertical bars indicate the treatment for the *L. perenne* crop planted (green), nitrate application at day 0 (blue), or defoliation of total aboveground vegetative biomass at day 28 (purple). Note no nitrate was added to Monocrop 1 columns

The analysed data from four types of column treatment are shown in Fig. 3. ‘No crop’ graph shows NO_3_^−^ application at day 0–6 with NO_3_^−^ detected by an increase in top sensor response (yellow plot). From day 2–4, NO_3_^−^ was detected in the middle portion of the column (middle sensor, orange plot), with a peak detected at days 20–22. This indicated leaching through the soil profile and from day 30 onwards increased NO_3_^−^ was detected by the bottom sensor (brown plot). The change in NO_3_^−^ detected at 12 hourly intervals reflects the diurnal changes in columns. In ‘Monocrop 1′ no NO_3_^−^ application was detected in any level, which is expected with no KNO_3_ added to this column. By the end of the experiment the above-ground vegetative biomass was low, as shown in Table 2, at only 5.7 ± 0.7 g, showing the *L. perenne* did not have optimal NO_3_^−^ supply for growth.

**Table 2:**
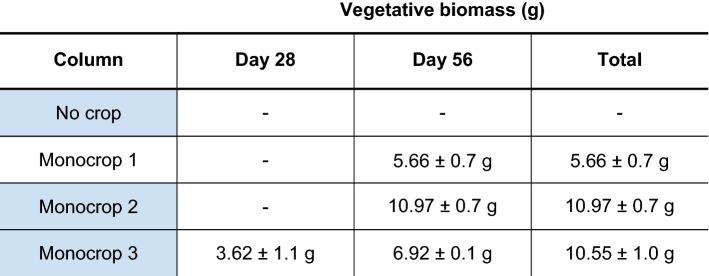
Total vegetative biomass for *Lolium perenne* monocropping experiment

Vegetative biomass is indicated in g for each column at day 28 (if defoliation occurred) and day 58. Total vegetative biomass is also included. Each value is a mean measurement across experiments, with standard deviation. Shading in column indicates nitrate application (blue). No biomass measurement is indicated with ‘–’

Figure 3 shows that for ‘Monocrop 2′ and ‘Monocrop 3′ a NO_3_^−^ application was detected at days 0–6 by the top sensor response (yellow plot), but both these columns were quickly depleted in NO_3_^−^ with little increase shown for middle sensor detection. This indicates that the NO_3_^−^ application was taken up by *L. perenne*, also inferred from the higher vegetative biomass measurements of 10–11 g compared with ‘Monocrop 1′ (Table 2). ‘Monocrop 2′ and ‘Monocrop 3′ differed in that defoliation occurred in the latter at day 28. An increase or ‘burst’ of detected NO_3_^−^ was detected by the middle sensor for the defoliation column only (orange plot).

For each plot, the standard error of the mean showed relatively low variation between column experiments. Column experiments allow researchers to carry out multiple repeats of experiments with defined parameters to generate reproducible data relating to plant-soil interactions. Soil columns allow for environmental fluctuations such as diurnal changes and N leaching through the soil profile to be studied more easily when compared to plants in the field. Data from media-based systems are less relevant to agriculture as they are often sterile and are usually in controlled environments for light, temperature and humidity. Hydroponics require regular changing of hydroponic solutions, with nutrients being replenished but soil physical and microbiological interactions are absent. Soil columns allow some control and standardisation of the environment and less heterogeneity when compared to field conditions, including microbial populations, light and temperature changes, and soil moisture levels. Although these environments may have less severe fluctuations than field trials, the columns allow for standardised measurements in laboratory settings which is more relevant than other systems. Soil temperature at depth in columns can vary more than occurs in the field. Standardisation is important for gaining meaningful data in a relatively short time compared to field trials investigating the effect of different management practices on NO_3_^−^ leaching. This is especially true for high intensity, temporary grasslands like those used by forage growers as there is a lack of evidence to suggest how different practices affect leaching in this environment, especially when compared to permanent pastures. By using soil column systems with in situ monitoring at three depths it is possible to see leaching in real-time in the ‘No crop’ columns. This movement of NO_3_^−^ through the soil profile was not shown in ‘Monocrop 2′ or ‘Monocrop 3′ suggesting leaching is ameliorated by the uptake of N by roots and can be used to measure the efficiency of root systems to acquire NO_3_^−^. The data suggests that *L. perenne* is cultivated efficiently using present forage crop practices with low levels of leaching.

### Increased NO_3_^−^ detected at mid-column depth following defoliation

To assess the detected NO_3_^−^ changes in more detail, plots showing each column depth separately are presented (see Additional file 1: Figures S2–S6). Additional file 1: Figure S2 shows graphs of independent levels for ‘No crop’ and ‘Monocrop 1′. These graphs show the statistically significant differences in column levels of detected NO_3_^−^. In addition, conventional soil water testing of drainage hole leachate (black diamonds on brown plot), showed good agreement with bottom sensor NO_3_^−^ measurements.

Graphs showing separate plots for each level for ‘Monocrop 2′ and ‘Monocrop 3′ are shown in Additional file 1: Figure S3. These show there were no statistically significant different periods in NO_3_^−^ measurements between sensors in either top or bottom levels throughout the 62 days of the experiment. On the other hand, the middle level transient increase in NO_3_^−^ following defoliation in ‘Monocrop 3′ is shown to be statistically significant (orange plot), between days 39–48 (p < 0.05). This middle level ‘burst’ reduces from day 48. Conventional soil water testing of drainage leachate, (black diamond plots), show good agreement with bottom sensor data.

**Fig. 4:**
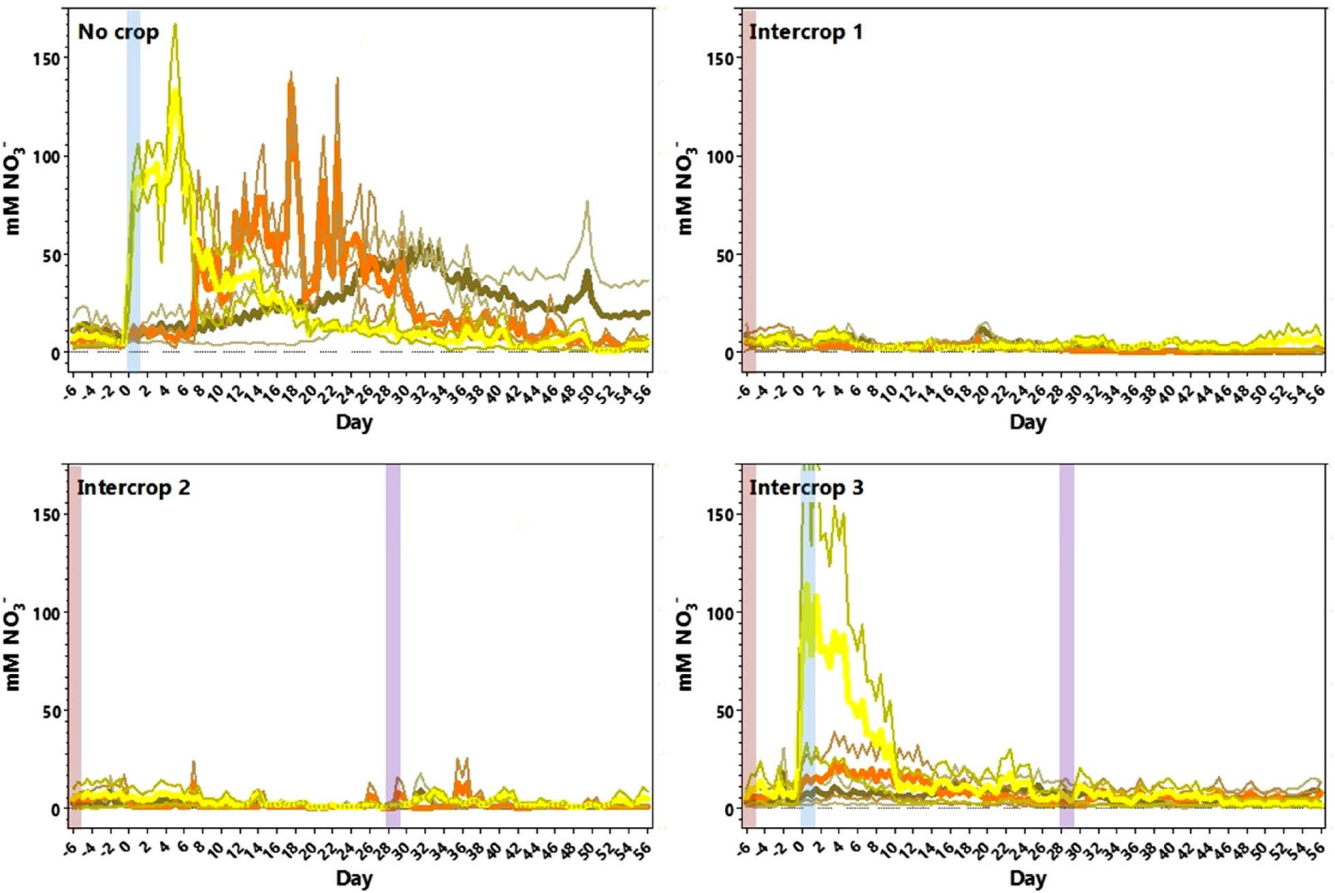
*Lolium perenne* and *Medicago sativa* intercrop column experiment NO_3_^−^-selective sensor data. Mean data (4 columns) are shown for each treatment as indicated in Table 3 as the thickest coloured lines. NO_3_^−^-selective sensor data are shown for top (yellow), middle (orange), and bottom (brown) depths in the columns. Data are the 12-hourly average of two experimental replicates in GraphPad Prism 7 (GraphPad Software Inc.), with standard error of the mean indicated with thinner lines of the same colour. Coloured vertical bars indicate the treatments for *L. perenne* and *M. sativa* crops (pink), nitrate application at day 0 (blue), or defoliation of total aboveground vegetative biomass at day 28 (purple)

By splitting NO_3_^−^ data for the different depths on columns, it is clear to see the wealth of information which can be provided by soil sensors for cost-effective analysis in columns in real-time (see Figs. 3, 4 and Additional file 1: Figures S2–S6). The NO_3_^−^-selective sensor data show similar measurements to conventional testing with less effort, which is important for precision agriculture [38]. Detail is also significantly increased, capturing changes in the soil profile and leaching movement; such data definition as 1-min sampling for 12 hourly means over a two-and-a-half-month period is not feasible with current methods. Thus NO_3_^−^-selective sensor can provide rich data describing rhizosphere processes and which is superior to current conventional testing methods.

The detected ‘burst’ in NO_3_^−^ found in the middle level of soil columns following defoliation of *L. perenne* from NO_3_^−^-selective sensors is very intriguing. It is possible that *L. perenne* released NO_3_^−^ as a stress response after cutting, or because of another process induced by defoliation. It is unlikely that the transient NO_3_^−^ ‘burst’ is an experimental artefact, as experimental replicates show standardised measurements with similar standard errors of the mean determined (Additional file 1: Figures S2 to S6). It could be argued that a reduction in NO_3_^−^ uptake was expected due to removal of vegetative tissue and this was observed for *L. perenne* in hydroponics [42]. In hydroponic systems it has been suggested that N can be released by roots at ~ 5.1–6.1% of total plant N storage under normal conditions [43]; however in solution culture it is difficult to test an individual region of the root.

Following defoliation in hydroponics it is well-documented that the remnant vegetative tissue preferentially takes up more carbon than N [44], in order to restore the C:N ratio of the tissue due to substantially decreased photosynthetic rate [45]. Total N reserves stored as vegetative storage proteins in roots and stem bases have been found to be rapidly degraded after defoliation [46]. Additionally, increasing defoliation of *Lolium* has led to decreased N uptake and increased plant N remobilization in hydroponic systems [47], and shoot tissue appears to be required for whole plant NO_3_^−^ reduction in grasses [48]. Moreover, remobilization of plant C-containing compounds in the leaf is shown to be coordinated with N availability to the root [49]. However, as mentioned previously, hydroponic systems cannot be used to assess specific levels of these compounds in roots or soil. It is likely that the detected NO_3_^−^ increase following defoliation in the column system disappears after 48 days when any available N may be re-taken up by the roots as vegetative growth has re-established photosynthesis in the shoot. Hydroponic systems may not record such changes due to the free diffusion of nutrients like soluble NO_3_^−^ through solutions. It is possible that other N-containing compounds are released by the roots as a stress response, and changes in amide and amino acid composition have been identified in *Lolium* xylem sap following defoliation [50], suggesting increased N assimilation [51, 52]. These N-compounds released by roots may be converted to NO_3_^−^ by rhizosphere microbes and demonstrated by changes in the rhizosphere microbiome before and after defoliation.

It is well-documented that grasses can release carbon exudates from roots in response to defoliation, including *Lolium* [22, 53, 54]. The complex carbon release profiles change depending on developmental stage or defoliation [55], and it is likely that such carbon exudates are linked to the measured increase in NO_3_^−^ in the middle level of soil column experiments reported here. This may be mediated by microbial activity which would be absent in media-based or hydroponic systems. Carbon exudates are not only important growth substrates for bacteria, but some may provide host-specific recognition signals promoting nitrifying bacteria [56]. Rhizosphere microbiome analysis after grazing or defoliation of grass has identified more gram-positive bacteria and increased inorganic N pools [3, 24]. We would predict a large transient increase in the population of nitrifiers in the microbiome and this might be driven by an acidification of the rhizosphere [57]. Furthermore, NO_3_^−^ generated by microbial activity which is usually taken up by leafed plants may not be when defoliation occurs, thus causing an increase in rhizosphere NO_3_^−^. In addition, root uptake is linked to changes in transpiration rate [58], reducing plant N uptake after defoliation [47]. The NO_3_^−^-selectivity of the soil sensors may be an important factor to consider for the increase measured after defoliation, as a large transient production of nitrite (NO_2_^−^) by rhizosphere bacteria may be reported by the sensors. The NO_3_^−^/NO_2_^−^ selectivity factor is tenfold greater for NO_3_^−^ [59]. However, other anions like organic acids, such as malate that might be released by the roots, are unlikely to be a problem [60].

### Soil NO_3_^−^ profiles when *Lolium perenne* was intercropped with Medicago sativa

Intercropping experiments were carried out with *L. perenne* and the legume *M. sativa*, as detailed in Table 3. As before one column had ‘No crop’, and the others were labelled ‘Intercrop 1–3′. ‘Intercrop 1′ had no NO_3_^−^ application, ‘Intercrop 2′ had a defoliation step at day 28, and ‘Intercrop 3′ had a NO_3_^−^ application at day 0 and defoliation at day 28. NO_3_^−^-selective sensor data for each column is found in Fig. 4. ‘No crop’ graph showed a NO_3_^−^ leaching pattern similar to ‘No crop’ in the monocropping experiments with NO_3_^−^ detected at day 0–6 at the top sensors of the column (yellow plot), with detection in the middle sensors with a peak at day 18 (orange plot), and a slightly earlier detection by bottom sensors from day 26. This data indicates again that the NO_3_^−^ application had leached through the soil profile when no crop was present. For ‘Intercrop 1′, NO_3_^−^ application was not detected in any level, as found in ‘Monocrop 1′. Total vegetative biomass, as shown in Table 4, was however higher for ‘Intercrop 1′ compared to ‘Monocrop 1′ at 8.0 ± 2.0 g, suggesting an N increase in the presence of the legume. ‘Intercrop 2′ graph was like ‘Intercrop 1′ with no significant change in NO_3_^−^ measured before or after defoliation at any level. Total vegetative biomass for ‘Intercrop 2′ was also similar to ‘Intercrop 1′ at 8.7 ± 1.3 g., see Table 4.

**Table 3:**
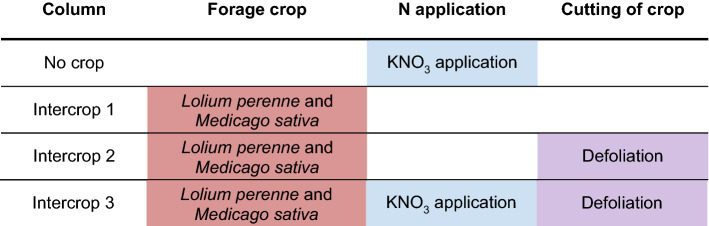
Soil column set-up for the *Lolium perenne* and *Medicago sativa* intercropping experiment

Columns underwent the management practice as colour coded as follows; pink for planting with 80:20 *L. perenne* cv. Aber Magic and *M. sativa* cv. Daisy seedlings, at a seeding rate of 43.7 kg ha^−1^ to match the forage industry standard; blue for day 0 nitrate application with KNO_3_ treatments equivalent to 57 kg ha^−1^; purple for day 28 total aboveground vegetative defoliation with biomass measurement. All columns with crops growing were cut on day 56 for harvesting the biomass total, and in the case of ‘Monocrop 3′ this new growth mass was added to the day 28 biomass measurement

**Table 4:**
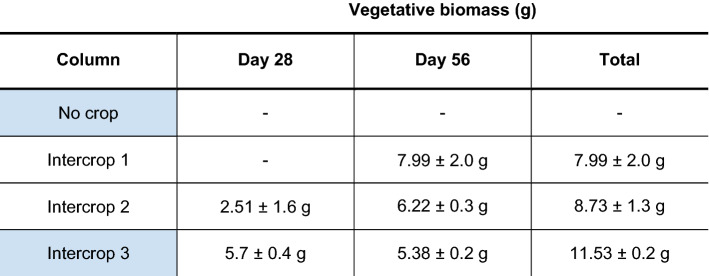
Total vegetative biomass for *Lolium perenne* and *Medicago sativa* intercropping experiment

Vegetative biomass is indicated in g for each column at day 28 (if defoliation occurred) and day 58. Total vegetative biomass is also included. Each value is a mean measurement across experiments, with standard deviation. Shading in column indicates nitrate application (blue). No biomass measurement is indicated with ‘–’

‘Intercrop 3′ in Fig. 4 was most similar in management practice to ‘Monocrop 2′ and ‘Monocrop 3′ treatments and the NO_3_^−^-selective sensor data is shown in Fig. 3. The NO_3_^−^ application was detected by the top sensors at the beginning or the experiment and quickly depleted with little detection of NO_3_^−^ by the middle level sensors from day 6. Total vegetative biomass measurement was also similar for ‘Intercrop 3′ and ‘Monocrop 2–3′, see Tables 2 and 4. However, despite ‘Intercropping 3′ undergoing defoliation at day 28, no NO_3_^−^ increase was detected by the middle sensors in contrast to the ‘Monocrop 3′ experiments.

### Transient NO_3_^−^ increase was not detected after defoliation in intercropped columns

To assess the NO_3_^−^ sensor data in more detail, individual sensor data plots were produced for column depth separately. Additional file 1: Figure S4 shows the graphs for ‘No crop’ and ‘Intercrop 1′, with statically significant differences indicated for detected NO_3_^−^ as described above. Conventional soil water testing of drainage leachate (black diamonds on brown plot), again showed good agreement with lowest depth NO_3_^−^ sensors measurements.

In monocrop conditions, upon defoliation of *L. perenne* NO_3_^−^ sensors detected a transient increase in NO_3_^−^ in the middle sensor region of soil columns and thus the roots (see Additional file 1: Figure S3). Additional file 1: Figure S5 shows intercropping conditions for separate column levels for defoliated *L. perenne* grown alongside *M. sativa*. Here in the intercropping experiment no transient NO_3_^−^ increase or ‘burst’ is evident at any depth of the column. Data for ‘Intercropping 2′ and ‘Intercropping 3′ suggests little evidence of a NO_3_^−^ ‘burst’ following defoliation, regardless of a NO_3_^−^ application being present or absent. Moreover, conventional soil water testing of drainage leachate again showed agreement with bottom NO_3_^−^-selective sensor data, although ‘Intercrop 3′ was slightly higher.

Furthermore, separate data for ‘Monocrop 3′ with ‘Intercrop 3′ were compared (see Additional file 1: Figure S6). These plots only showed a significant difference in the top and bottom level NO_3_^−^-selective sensor measurements for a short period at the start of the experiment. This may result from the smaller number of replicates in these intercropping experiments. Most strikingly was the difference between experiments at the middle depth where statistically significant differences were found between plots (orange plot). Despite applying the same management practices, a transient increase in NO_3_^−^ was not detected following defoliation in ‘Intercrop 3′. This result was statistically significant when compared to ‘Monocrop 3′ between days 34–44 (p < 0.05).

As the middle region NO_3_^−^ ‘burst’ was not found for ‘Intercrop 3′ with *L. perenne* grown alongside *M. sativa*, it is possible that growing the legume has caused a change in the microbial populations that altered N dynamics in the soil. It is known that greater rhizosphere microbiome diversity is found for legumes when compared with grasses [61, 62]. Alternatively, the difference with intercropping may be due to variation in root architecture of legumes. The *L. perenne* root density is probably decreased in the intercropping column when compared with monocropping and it may be possible that the transient NO_3_^−^ release is diluted by the mixed root population and can no longer be detected. Nonetheless, the root density is only slightly reduced as the 80:20 plant mix means that only a few *M. sativa* plants are found in each column. Moreover, this explanation would still require that the legume root responds differently to the grass after defoliation. Defoliation causes changes in transpiration rates in many plants and this effect may be species dependent, so having more species may change how roots interact with the soil [63]. Work comparing grass and legume root responses to defoliation has suggested similar below ground responses [23, 46, 52, 64], but perhaps more investigation in required, although specific species are known to vary in their root branching shapes [65]. Plant root idiotypes having slightly different patterns are important for breeders [66] and, for forage crops, the effect of defoliation on root physiology including the microbiota is likely to be a key trait [67]. The differences found between grasses and legumes, comparing a long tap root in the former to a more branched legume root structure in the latter [68–70], may influence the soil N profile changes following defoliation in intercropping systems. Interestingly in mixed clover and grass swards defoliation also caused a change in the composition of microbial populations, although there was no significant effect on microbial activity [71], but our data shows there was no commitment release of NO_3_^−^ (see Fig. 4).

## Conclusions

These NO_3_^−^-selective sensors can be built in laboratories quickly and cheaply (Additional file 1: Figure S1) and they can measure in real time the available soil water NO_3_^−^ after calibration with known NO_3_^−^ concentrations. Furthermore, these measurements that show good agreement with conventional testing methods (Fig. 2, Additional file 1: Figure S2–S6). The sensors offer the advantage of the rapid reporting of soil available nitrate offering a method that is much easier to use when compared with lab chemical assays. However, the sensor measurements may be influenced by chemical interference and this is more likely to be a problem at low concentrations near the detection limit (0.5 mM nitrate [59, 60]). The sensors can be deployed in soil columns to simulate conditions in the field. The transient release of NO_3_^−^ in the middle soil column region following defoliation of *L. perenne* was consistent across experiments and was revealed by using the soil NO_3_^−^ sensors. The rhizosphere transient NO_3_^−^ release we have observed may not be a problem for forage growers, as plants seem to uptake the available NO_3_^−^ with little evidence of leaching from the profile. Furthermore, the lack of a NO_3_^−^ ‘burst’ when *L. perenne* was intercropped with *M. sativa* provides evidence for possible advantages for forage crop recovery after cutting although the increase in biomass with intercropping was relatively small (mean values of 11.5 g vs 10.6 g; Tables 2 and 4). Differences between monocropping and intercropping forage systems have already been identified as contributing to above- and below-ground species diversity, significantly affecting soil erosion in studies of permanent pastures [63]. This work used the soil column system to monitor rhizosphere NO_3_^−^ but other types of nutrient-selective sensors could be built in a similar way by altering the ion-selective membrane used [59, 72]. The use of the soil column and sensor system allows the dynamic monitoring of changes in soil profile nutrient concentrations that can be used to screen crop genotypes with improved rhizosphere traits that reduce leaching losses.

## Materials and methods

### ***NO***_***3***_^***−***^*** -selective sensor construction***

NO_3_^−^-selective sensors were constructed as described previously [60, 72] using the construction scheme found in Additional file 1: Figure S1. Sensors used 1.25 mL pipette tips (Starlab, Milton Keynes, UK), with tips silanized to a depth of approximately 1 cm with Repelcote™ (Dow Corning, Gillingham, UK). Two membrane solutions were prepared in 2 mL final volume of tetrahydrofuran solvent (Millipore, ≥ 99.9% 1,081,100,500), as follows with chemicals from Sigma-Aldrich unless specified.

- NO_3_^−^-selective membrane containing 12 mg tridodecylmethylammonium NO_3_^−^, 2 mg methyltriphenylphosphonium bromide, 46 mg poly(vinyl chloride) (PVC), 10 mg nitrocellulose (Amersham Hybond ECL, RPN2020D, 0.45 μM, 200 × 200 mm, GE Healthcare), and 130 mg 2-nitrophenyl octyl ether.
- Reference membrane containing 2 mg potassium tetrakis(4-chlorophenyl)borate, 45 mg polyethylene glycol 3500 and 10 mg nitrocellulose.

Membrane solutions were covered with foil, capped, and sealed with parafilm, then shaken at 150 rpm overnight to ensure reagents were dissolved thoroughly. Silanized tips were then dipped into one of the membrane solutions to a depth of approximately 2 cm. Tips were dried in a fume hood for 48 h to produce a thin (approximately 2–3 mm) membrane. Two backfill solutions were prepared in 200 mL dH_2_O.

- Ion-selective backfill containing 2.202 g KNO_3_ and 1.49 g KCl.
- Reference backfill containing 3.12 M KCl, 20 mg AgCl_2_, 1.8 g NaCl, and 0.18 g naphthol green B.

One mL of the backfill solution was loaded into the top of the corresponding membraned tip, air bubbles were displaced, and a sensor of each type paired together. Sensor cables were prepared by stripping ~ 1 cm from each end of 1.5 m lengths of wire (RS Components Ltd.), with one end clamped with ~ 7 cm of Ag wire (99.9%, Palmer Metals, Coventry UK), coated in 50 mM KCl. This end was threaded through an earplug (RS Components Ltd.) and inserted into the backfilled tip using a disposable needle to displace air. Sensors were secured with black cable ties (RS Components Ltd.) and secured in pairs with 2 × 5 cm strips of parafilm (Slaughter Ltd, Basildon, UK). The final sensors and their performance in comparison to a conventional N assay are shown in Fig. 2.

### ***NO***_***3***_^***−***^***-selective sensor calibration***

Sensor pairs (NO_3_^−^-selective and reference) were connected to GP2 loggers (Delta-T Devices Ltd., Cambridge, UK), where ion-selective sensors were (+) channels and paired reference sensors were (−). A calibration programme was installed using DeltaLINK 3.6.2 (Delta-T Devices Ltd.) for ‘Voltage, not powered’ and circuit detection and power channel disabled. A calibration using eight nitrate solutions revealed an identical calibration curve to those previously reported for glass microelectrodes [59, 60]. This calibration could be fitted with a simplified Nicolsky–Eisenman equation to show the same detection limits (0.1 mM) and ion-selectivity coefficients like those reported previously [59, 60]. The calibration was simplified to a linear fitted line for the soil measurements and sensors were placed into solutions of 300, 30, 3 and 0.3 mM KNO_3_ sequentially for a minimum of 5 min each. The electrical potential of sensors was measured and recorded (mV), and the mean calculated for the final 1 min period in each concentration. A linear regression was fitted for individual sensors alongside the known concentration providing a calibration Eq. (1) for each sensor using Excel® 2016 (Microsoft®).

1 $$mV=\left(m\times log_{10}N{O}_{3}^{-}mM\right)+c$$

Sensors with slope factor ‘m’ outside of 45–55 mV were considered not useable and reconstructed. Useable sensors were stored in solutions of 100 mM KNO_3_ until use. A Delta-T SM300 soil moisture and temperature sensor was also connected into an available channel of one logger.

### NO_3_^−^-selective sensor measurements and data analysis

Sensors were placed in columns with care to limit disturbance to tip membranes. One to three sensors were placed at each level and outputs (mV) were recorded at 1 min intervals. Sensors ran in columns for a week before experiments began to check all sensors were working and were replaced as needed. Sensors could be conveniently removed from the column, recalibrated and if necessary, easily replaced. Waterproof tape covered the sensor hole for a few hours during this process. Most sensors showed very similar accuracy and calibration and were successfully recalibrated at end of the experiment (62 days).

A laboratory temperature slope coefficient, included in the analysis to compensate for temperature changes in glasshouse between experiments, was derived from the Nicolsky-Eisenmann relationship [59]. Experimental mV outputs were compared to theoretical calculated values across temperatures from 8 to 35 °C. A linear coefficient of compensation was calculated and included in analysis of NO_3_^−^ mM (2):

2 $$Temperature\, compensation=\left(0.405\times^\circ{C}\right)+93.6$$

For each experiment a 6-d resting period (day − 6 to 0) was included with sensor data recorded and watering of soil columns every 2–4 days with dH_2_O. The experiment then ran from 0 to 56 days. At the end of experiments sensors were recalibrated and, if individual sensors had changed significantly from their first calibration (slope ‘m’), then all their recorded data was removed from subsequent analysis. The arithmetic means for 12 hourly periods between 0 and 12 h were calculated using Excel® 2016. Data were plotted in GraphPad Prism 7 (GraphPad Software Inc.) and analysed in GenStat® 18th Edition (VSN International) to determine statistical significance between columns using student T-tests. A repeated analysis using ANOVA in RStudio (RStudio, Inc.) was used to validate the analysis.

### Soil column experiments

Four PVC opaque columns (height = 50 cm, inside diameter = 15.4 cm, wall thicknesses of 0.5 cm) with 5 drainage holes at base (made by John Humble, John Innes Centre Workshop, Norwich, UK) were filled with silty clay soil (sourced from Church farm, John Innes Centre, 52°37′ 59.8836′′ N 1°10′ 46.3440′′ E). Columns included holes for sensors at three levels, top (1 cm depth), middle (25 cm depth) and bottom (49 cm depth), see Fig. 1. Water-holding capacities for soil columns were determined, and columns watered every 2–4 days with dH_2_O to a similar capacity to promote nutrient flow through the profile. The experiments were conducted in the greenhouse using the locally collected soil and analysis before the experiment revealed NO_3_–N 101.9 mg kg^−1^, NH_4_–N 2.6 mg kg^−1^, Olsen P 9.55 mg kg^−1^, pH 8.07, organic matter 2.5 g kg^−1^. There were four experimental replicate columns for each treatment.

At day 0 the experiment began with the column experimental design summarised in Table 1. KNO_3_ treatments were 57 kg ha^−1^, equivalent to the rate of first application for UK forage crop cultivation [73]; this was equivalent to 10.76 g KNO_3_ in 1 L for each column, or a dH_2_O control. Seeds of *Lolium perenne* cv. Aber Magic were kindly provided by the Genebank at The Institute of Biological, Environmental and Rural Sciences (Aberystwyth University, Aberystwyth, Wales, UK). Seeds were surface-sterilised with ethanol 70% (v/v) at a seeding rate of 43.7 kg ha^−1^ or 0.83 g per column. These were germinated on paper towels for 6 d before transplantation into columns. For defoliation, after 4 weeks the whole vegetative tissue in one column was cut to the soil level to simulate cropping. This tissue was oven dried overnight at 55–60 °C and biomass recorded. For all columns at 8 weeks the total vegetative tissue was cut and again biomass recorded. The experiment was repeated four times. By the end of the experiments the roots of all the forage crops had grown throughout the column.

For one experiment conventional soil water sampling was carried out using soil water samples collected from the base of columns using a mini suction lysimeter (10 Rhizon SMS, Rhizosphere Research Products B.V., Wageningen, The Netherlands). Soil water samples were collected every 1–4 days with analysis at the end of experiment.

Column experiments were repeated as above for intercropping experiments. The experimental design is shown in Table 3 and largely matched that of monocropping experiments. Intercropping columns had a seeding rate mix of 80:20 grass: legume simulating the planting density used by the UK forage industry. This used *L. perenne* at 0.66 g per column and *Medicago sativa* cv. Daisy (DLF Forage Seeds, DK, provided by Dengie Crops Ltd., UK), at 0.08 g per column. All other practices matched monocropping experiments, with soil water for conventional soil water analysis taken from drainage holes from one experiment. The whole experiment was repeated twice.

### Spectrophotometric Griess determination of NO_3_^−^ in soil eluates

A reduction-diazotisation reagent was prepared by adding the following solution A to solution B. Solution A was 400 mg VCl_3_ to 50 mL HCl 1.0 M, with gentle shaking until dissolved. Solution B was 200 mg of sulphanilamide and 10 mg *N*-(1-naphthyl)ethylenediamine dihydrochloride in 400 mL dH_2_O [28].

Standard solutions of KNO_3_ were prepared in 10 mL KCl 2.0 M for NO_3_^−^ concentrations of 0, 0.2, 0.4, 0.6, 0.8, 1.0, 1.2, 1.4, 1.6, 1.8, and 2.0 μg/mL. To 3.5 mL cuvettes (Sarstedt Limited, Nümbrecht, Germany), 1 mL of standard or 1 mL of water sample was added, then 800 μL of the above reduction-diazotisation reagent. Samples were incubated for 20 h at room temperature and their absorbance at 540 nm measured. A calibration regression was calculated using measurements for standards, with R^2^ of ≥ 0.98. These data were used to give a linear equation for calculating NO_3_^−^ concentrations in soil water samples, corrected by the dilution factor of the KCl solution.

## Supplementary Information

**Additional file 1: Figure S1.** Schematic diagram of NO_3_^−^-selective sensor construction. **Figure S2.** *Lolium perenne* monocrop column experiment NO_3_^−^-selective sensor data for ‘No crop’ and ‘Monocrop 1′. **Figure S3.** Monocrop column experiment NO_3_^−^-selective sensor data for ‘Monocrop 2′ and ‘Monocrop 3′. **Figure S4.** *Lolium perenne* and *Medicago sativa* intercrop column experiment NO_3_^−^-selective sensor data for ‘No crop’ and ‘Intercrop 1′. **Figure S5.** Intercrop column experiment NO_3_^−^-selective sensor data for ‘Intercrop 2′ and ‘Intercrop 3′. **Figure S6** Column experiment NO_3_^−^-selective sensor data for ‘Monocrop 3′ and ‘Intercrop 3’.

## Data Availability

The images and datasets used and analyzed during the present study are available from the corresponding author on reasonable request.

## Abbreviations

ANOVA: Analysis of variance
PVC: Poly(vinyl chloride)
