# Supplementary material for: Real-time monitoring of rhizosphere nitrate fluctuations under crops following defoliation
Source: Plant Methods. 2021 Jan 30;17:11. doi: 10.1186/s13007-021-00713-w (PMC7847023; doi:10.1186/s13007-021-00713-w)
Supplement: Supplementary file 1 — Additional file 1: Figure S1. Schematic diagram of NO3−-selective sensor construction. Figure S2. Lolium perenne monocrop column experiment NO3−-selective sensor data for ‘No crop’ and ‘Monocrop 1′. Figure S3. Monocrop column experiment NO3−-selective sensor data for ‘Monocrop 2′ and ‘Monocrop 3′. Figure S4. Lolium perenne and Medicago sativa intercrop column experiment NO3−-selective sensor data for ‘No crop’ and ‘Intercrop 1′. Figure S5. Intercrop column experiment NO3−-selective sensor data for ‘Intercrop 2′ and ‘Intercrop 3′. Figure S6 Column experiment NO3−-selective sensor data for ‘Monocrop 3′ and ‘Intercrop 3’. [file 13007_2021_713_MOESM1_ESM.pdf]

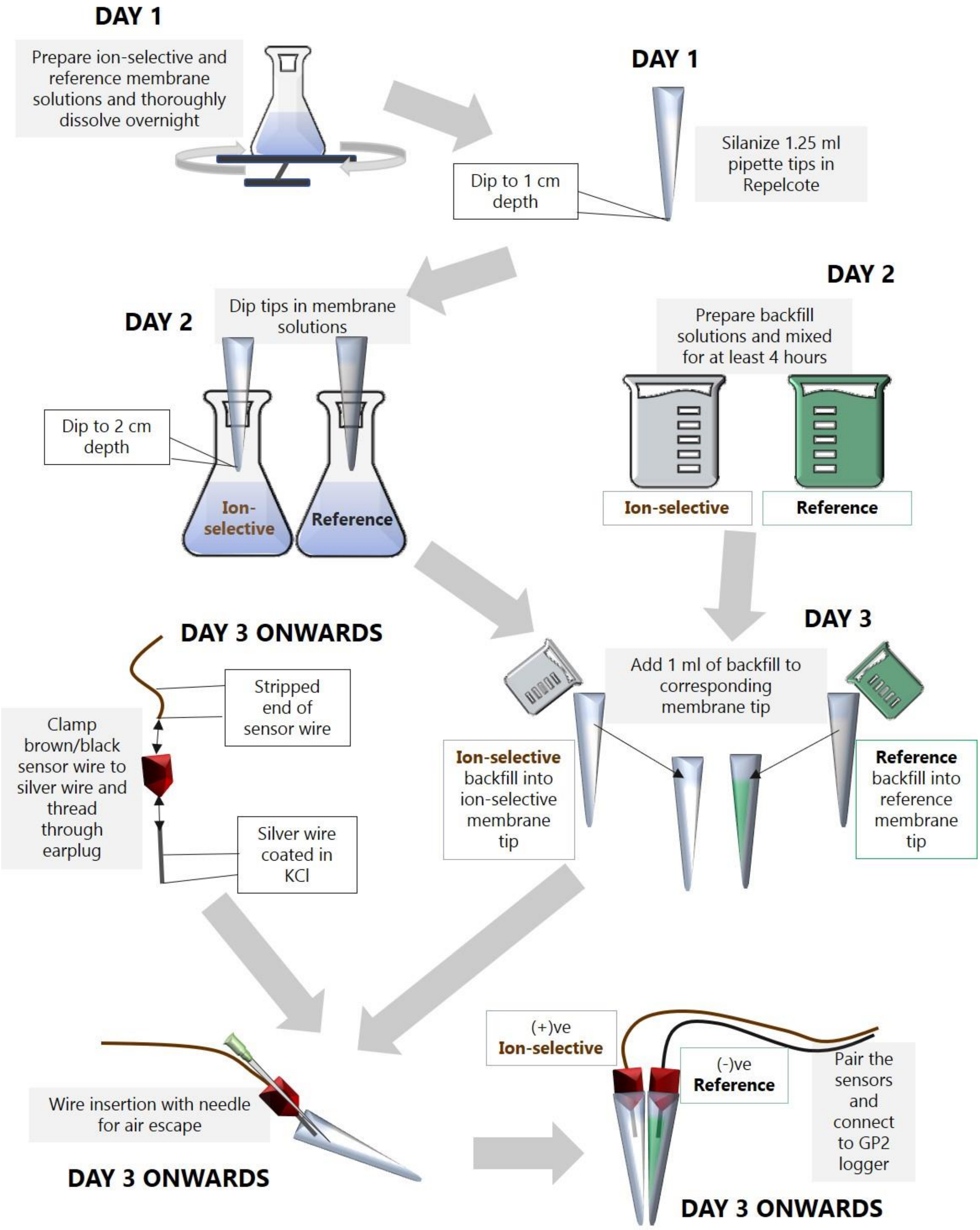

Supplementary Figure S1. Schematic diagram of NO_3^--selective sensor construction.

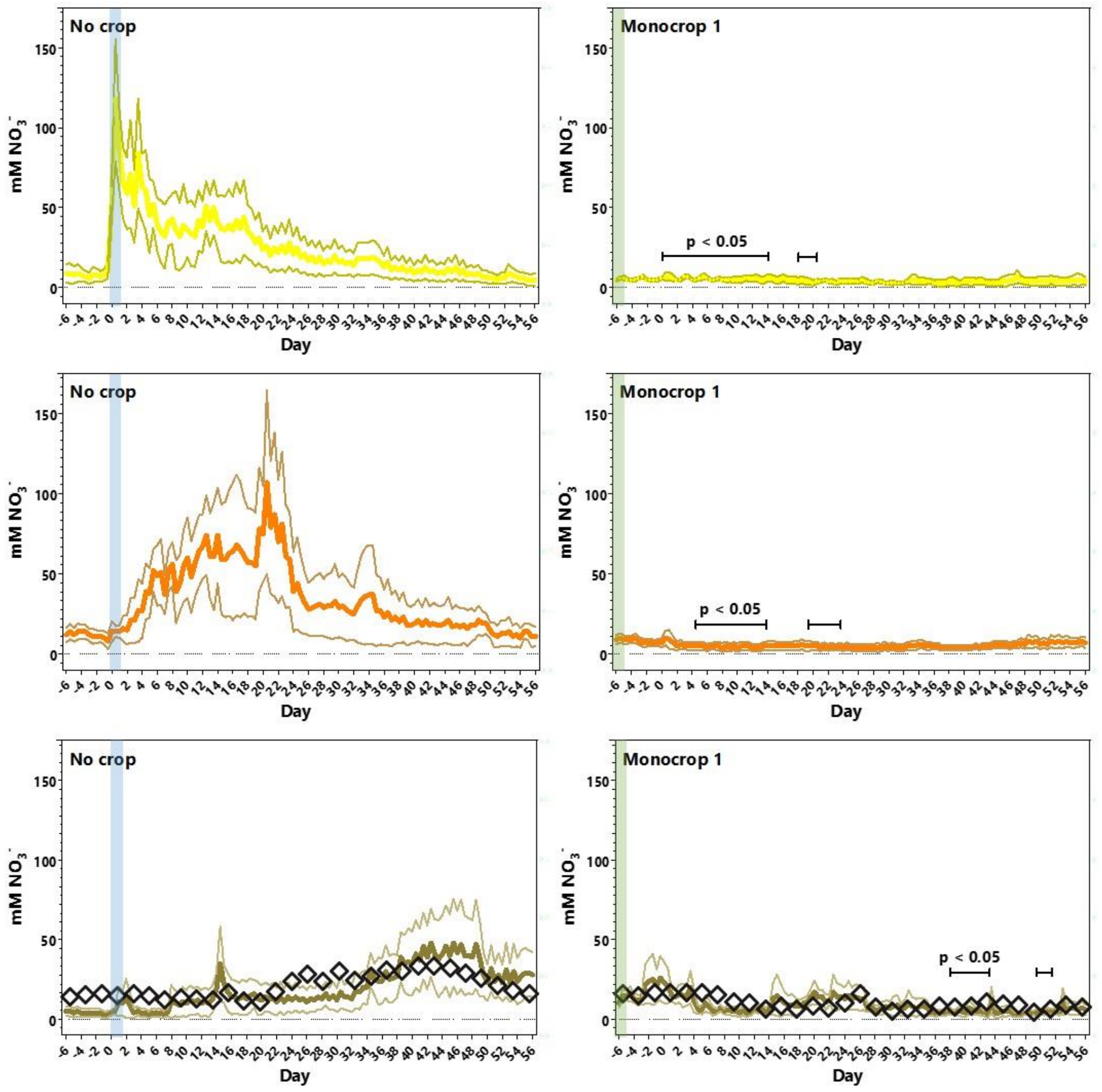

Supplementary Figure S2. *Lolium perenne* monocrop column experiment NO_3^--selective sensor data for 'No crop' and 'Monocrop 1'.

Columns treatments are described in Table 1. NO_3^--selective sensor data are shown independently for top (yellow), middle (orange), and bottom (brown) column levels. Data is the 12-hourly average of four experimental replicates plotted in GraphPad Prism 7 (GraphPad Software Inc.), with standard errors of the means indicated with thinner lines of a similar colour. Coloured vertical bars indicate management practice of *L. perenne* crop planted (green) and NO_3^- application at day 0 (blue). A student one-way t-test was carried out in Excel for 12-hourly timepoints between columns for each independent depth level. In the bottom level graph, the soil water from drainage holes

for one experiment was tested as leachate using a chemical assay and indicated by black diamond symbols.

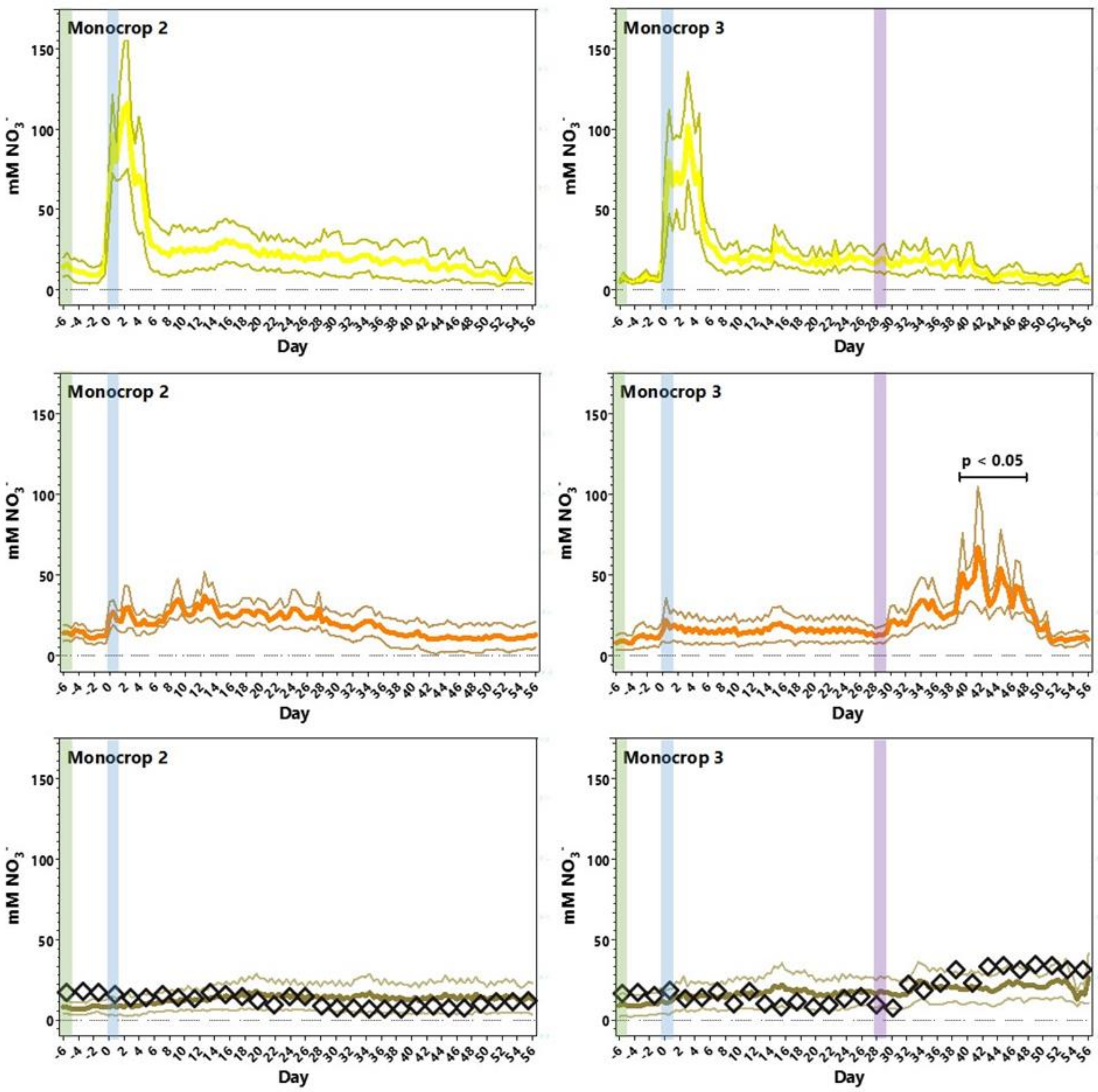

Supplementary Figure S3. Monocrop column experiment NO_3^--selective sensor data for ‘Monocrop 2’ and ‘Monocrop 3’.

Column treatments are described in **Table 1**. NO_3^--selective sensor data are shown independently for top (yellow), middle (orange), and bottom (brown) levels of columns. Data is the 12-hourly average of four experimental replicates plotted in GraphPad Prism 7 (GraphPad Software Inc.), with standard errors of the means indicated with thinner lines of a similar colour. Coloured vertical bars indicate management practice

of *L. perenne* crop planted (green) and nitrate application at day 0 (blue). A student one-way t-test was carried out in Excel for 12-hourly timepoints between columns for each independent depth level. In the bottom level graph, the soil water from drainage holes for one experiment was tested as leachate using a chemical assay and indicated by black diamond symbols.

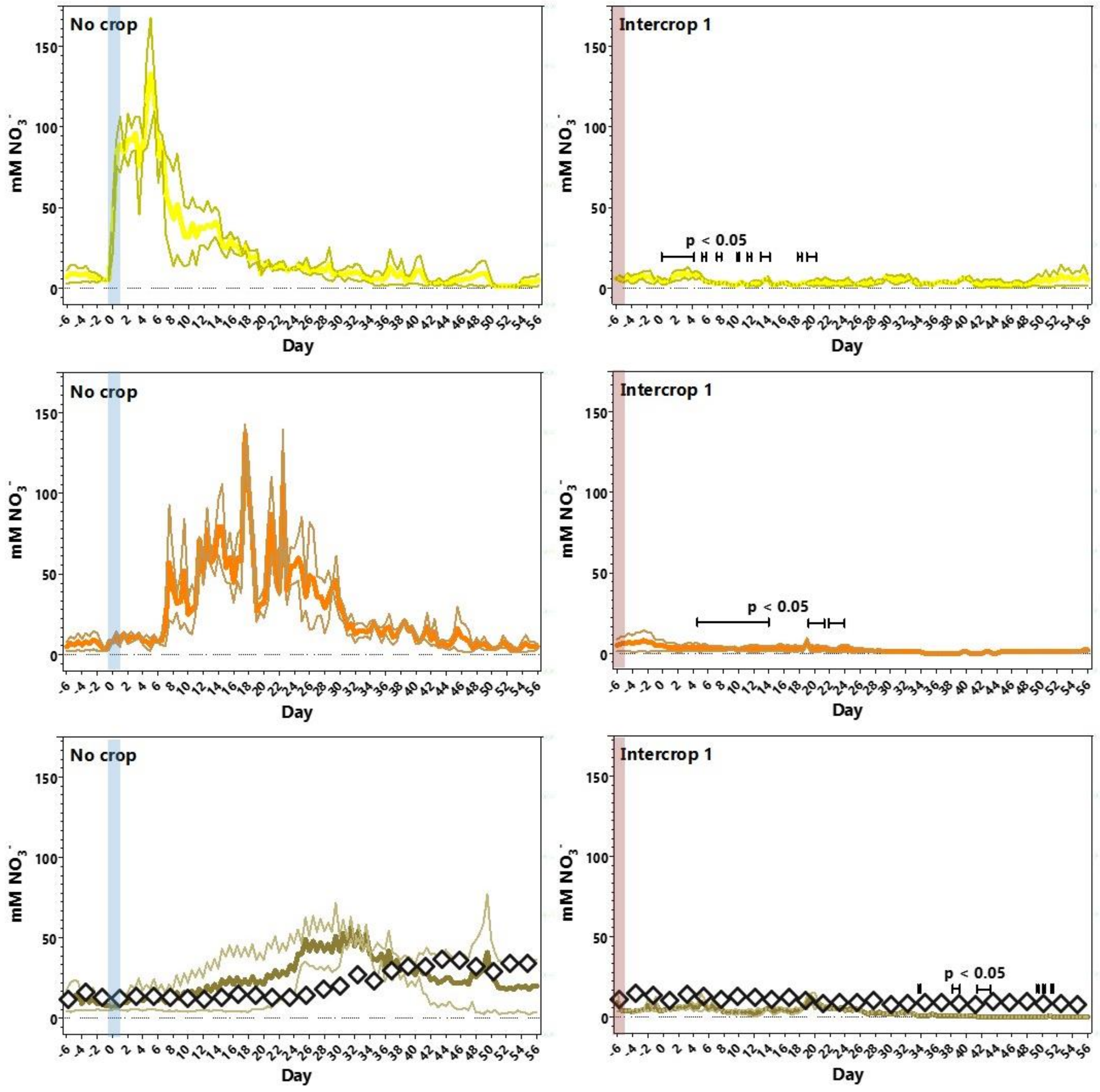

Supplementary Figure S4. *Lolium perenne* and *Medicago sativa* intercrop column experiment NO_3^--selective sensor data for 'No crop' and 'Intercrop 1'.

Column treatments are described in Table 3. NO_3^--selective sensor data are shown independently for top (yellow), middle (orange), and bottom (brown) levels of columns. Data is the 12-hourly average of four experimental replicates plotted in GraphPad

Prism 7 (GraphPad Software Inc.), with standard errors of the means indicated with thinner lines of a similar colour. Coloured vertical bars indicate management practice of *L. perenne* and *M. sativa* crop planted (pink) and NO_3^- application at day 0 (blue). A student one-way t-test was carried out in Excel for 12-hourly timepoints between columns for each independent depth level. In the bottom level graph, the soil water from drainage holes for one experiment was tested as leachate using a chemical assay and indicated by black diamond symbols.

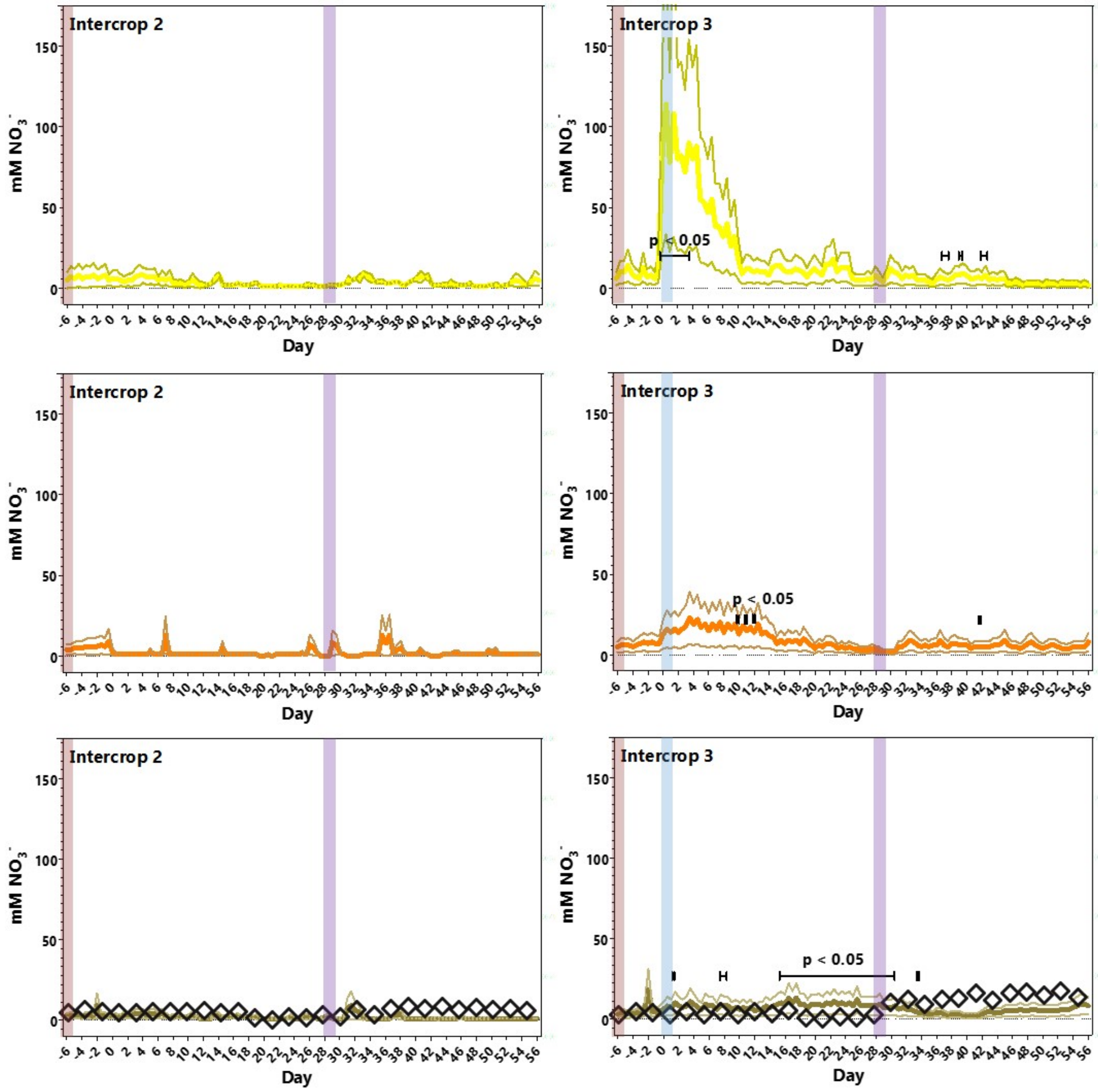

Supplementary Figure S5. Intercrop column experiment NO_3^--selective sensor data for 'Intercrop 2' and 'Intercrop 3'.

Column treatments are described in Table 3. NO_3^--selective sensor data are shown independently for top (yellow), middle (orange), and bottom (brown) levels of columns. Data is the 12-hourly average of four experimental replicates plotted in GraphPad Prism 7 (GraphPad Software Inc.), with standard errors of the means indicated with thinner lines of a similar colour. Coloured vertical bars indicate management practice of *L. perenne* and *M. sativa* crop planted (pink) and NO_3^- application at day 0 (blue). A student one-way t-test was carried out in Excel for 12-hourly timepoints between columns for each independent depth level. In the bottom level graph, the soil water from drainage holes for one experiment was tested as leachate using a chemical assay and indicated by black diamond symbols.

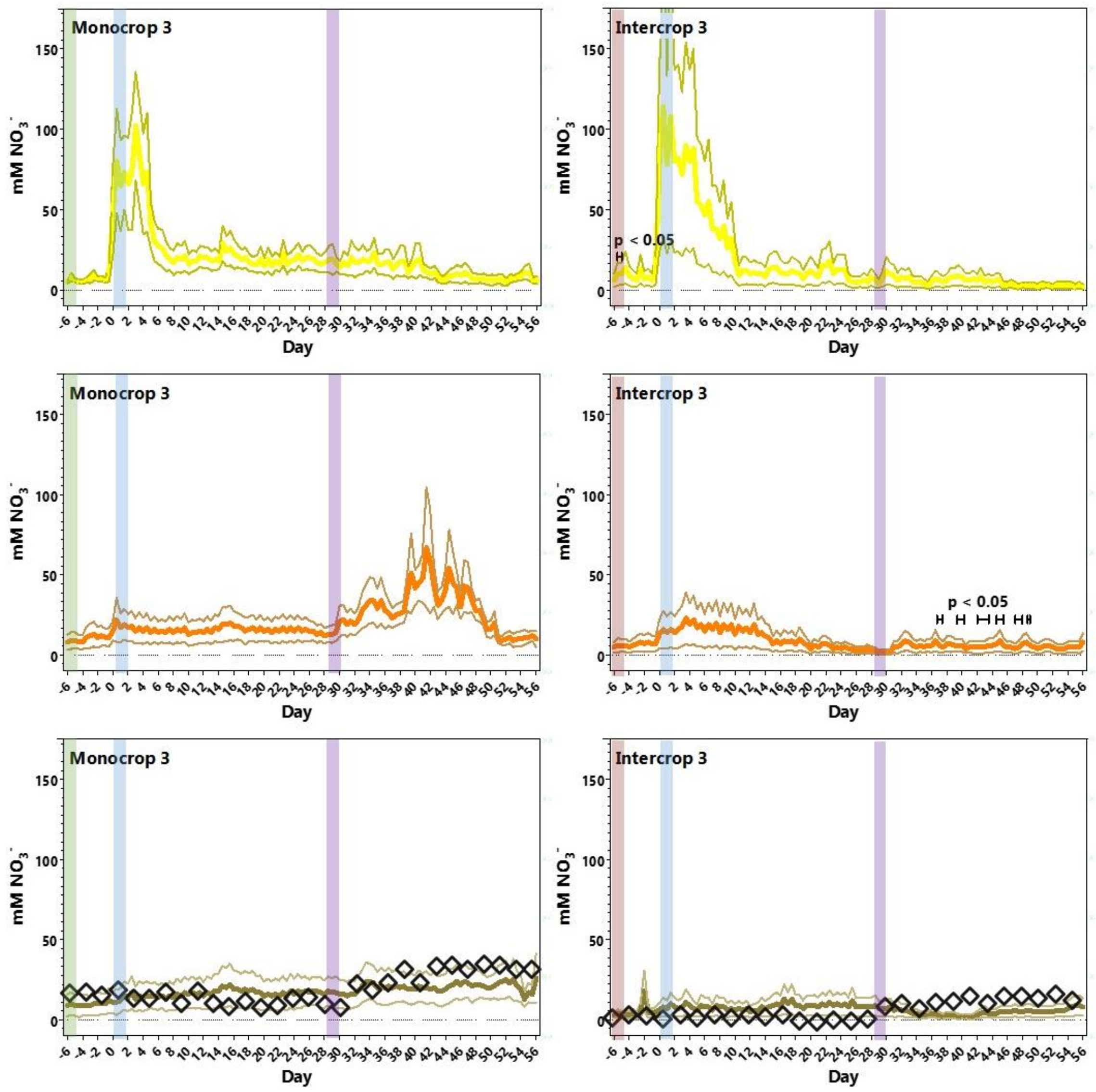

Supplementary Figure S6. Column experiment NO_3^--selective sensor data for 'Monocrop 3' and 'Intercrop 3'.

NO_3^--selective sensor data are shown independently for top (yellow), middle (orange), and bottom (brown) levels of columns. Data is the 12-hourly average of four experimental replicates plotted in GraphPad Prism 7 (GraphPad Software Inc.), with standard errors of the means indicated with thinner lines of a similar colour. Coloured vertical bars indicate management practice of *L. perenne* (green), or with *M. sativa* (pink) and NO_3^- application at day 0 (blue). A student one-way t-test was carried out in Excel for 12-hourly timepoints between columns for each independent depth level. In the bottom level graph, the soil water from drainage holes for one experiment was tested as leachate using a chemical assay and indicated by black diamond symbols.
